# Editorial: MacIntyrean virtue ethics for organizations, work and employment: What more and what else?

**DOI:** 10.3389/fsoc.2023.1145849

**Published:** 2023-03-28

**Authors:** Caleb Bernacchio, Geoff Moore, Angus Robson, Marta Rocchi

**Affiliations:** ^1^College of Business, California State University Monterrey Bay, Seaside, CA, United States; ^2^Business School, Durham University, Durham, United Kingdom; ^3^Newcastle Business School, Northumbria University, Newcastle upon Tyne, United Kingdom; ^4^DCU Business School, Irish Institute of Digital Business, Dublin City University, Dublin, Ireland

**Keywords:** organization studies, Alasdair MacIntyre, strategy, chronic moral injury, climate change, institutional logics, business ethics, practice-institution

A Research Topic on MacIntyrean virtue ethics for organizations, work and employment might seem to be so narrowly focused as to be of interest only to a very small group of scholars. But the audience for this collection of articles should be much wider. Why so?

First, Alasdair MacIntyre has been one of the leading moral philosophers of the 20th and early 21st centuries. His critique of modernity in general, and the Enlightenment project in particular, has been countered with his positive proposals to return to an ethic of virtue based principally on the work of Aristotle and Thomas Aquinas. His work is worth reading for its own sake.

Second, one remarkable feature of MacIntyre's work has been its application to an extraordinarily wide range of disciplines beyond philosophy itself, as evidenced in *Learning from MacIntyre* (Beadle and Moore, 2020). This extensive secondary literature is worth almost any scholar from almost any discipline exploring for application to their own work.

Third, and of specific relevance to this Research Topic, is the application of MacIntyre's work to organizations in general, and business organizations in particular, with its implications for work as participation within practices, meaningful work, and employment. This has an established place in the business ethics and organization studies literature, with interest extending well-beyond just MacIntyrean scholars. The contributions to date were summarized in Moore (2017). However, since this was published, there has been a considerable number of further publications in this area, and in 2016 MacIntyre published *Ethics in the Conflicts of Modernity*, with insights into personal and organizational ethics which, due to publication deadlines, could not be captured in Moore's book. Thus, for organizational scholars in particular, there is more to be said.

The goals of this Research Topic were, therefore, three-fold: first, to update the summary of the application of MacIntyre's work to organizations, work and employment that was included in Moore (2017)—“Where are we now?”; second, to invite critical reflections on this body of work—“What criticisms of the work to date does future research need to take into account?”; and third, to invite both general reflections and more specific research that develops the field—“What more and what else?”.

The six articles in this Research Topic cover these three goals. Burns takes us back to the philosophical roots of MacIntyre's work, and examines the distinction between “internal goods” and “external goods”. This then has implications for two of MacIntyre's other key concepts, “practices” and social “institutions”, and their inter-relationship. He argues that there is a largely unrecognized element of Platonism in MacIntyre's work.

Bernacchio and Couch also take us back to early work in the application of MacIntyre's notion of a practice to organizations. They argue, against the established understanding of organizations as “housing” a core practice and as practice-institution combinations, that business is itself a practice. Drawing on the strategy literature, they argue that business has an integrative function in relation to other practices in such a way as to enable mutual benefits. This might be seen, somewhat controversially, as an appropriation of the established understanding from a classical liberal perspective, and so is likely to lead to further debate.

By contrast, Bolade-Ogunfodun et al. draw on the established understanding of organizations as practice-institution combinations, and offer an empirical article based on an ethnographic study of a company following its acquisition. While offering further support to MacIntyre's critique of management, they extend previous work by introducing the concept of “practice-like” activities, exploring the extent to which they are vulnerable to institutional demands. They also give consideration to organizational culture, friendship and the common good in relation to such activities.

Abadal and Potts also draw on the established framework but extend it by developing an account of “chronic moral injury” in the workplace. Chronic moral injury occurs in “vicious” institutions which undermine both practices and the development of virtues. A case study of the biotech company Theranos provides a further empirical contribution to MacIntyrean studies, and demonstrates how chronic moral injury can occur, and its implications for practitioners.

While vicious institutions form a part of the existing literature, Moore develops the more common application of MacIntyre's work to the notion of virtuous organizations. Drawing on MacIntyre's *Ethics in the Conflicts of Modernity* (MacIntyre, 2016), with its focus on desire, consumption and human flourishing, the theoretical contribution proposes virtuous organizations as contributing to the way in which desires might be redirected and re-educated, leading to both individual and communal good. Taking the discussion in a direction which has not been addressed either by MacIntyre himself or in the literature that draws on his work, the article applies this extended theory of the virtuous organization to the issue of climate change. Contrasts with the degrowth movement are made, demonstrating the way in which a MacIntyrean approach is distinctive.

Finally, Chu's article contributes to the “what more and what else” of MacIntyrean studies in her consideration of neo-institutional theory and institutional logics—key topics in organization studies. The development of a typology of goods based on MacIntyre's work is used to explore how different institutional orders (market and family) and their logics place different emphasis on different types of goods. Neo-institutional theory's deficiency in relation to morality is then critiqued by drawing on these aspects of MacIntyre's work.

There is, therefore, much for business ethics and organization scholars to ponder on here, and potentially to incorporate into their own research. Doubtless, therefore, the articles in this Research Topic will not be the last word on the matters to which MacIntyre's work directs us.

## Author contributions

All authors listed have made a substantial, direct, and intellectual contribution to the work and approved it for publication.
